# Changes in lymphocyte subsets pre- and post-particle radiotherapy in head and neck bone and soft tissue tumors

**DOI:** 10.3389/fonc.2025.1543718

**Published:** 2025-06-16

**Authors:** Haojiong Zhang, Jing Gao, Jiyi Hu, Weixu Hu, Qingting Huang, Lin Kong

**Affiliations:** ^1^ Department of Radiation Oncology, Shanghai Proton and Heavy Ion Center, Fudan University Cancer Hospital, Shanghai, China; ^2^ Shanghai Key Laboratory of Radiation Oncology, Shanghai, China; ^3^ Shanghai Engineering Research Center of Proton and Heavy Ion Radiation Therapy, Shanghai, China

**Keywords:** bone and soft tissue tumors, head and neck cancer, peripheral lymphocyte immunophenotype, particle radiation, radiation immunity

## Abstract

**Purpose:**

Bone and soft tissue tumors present unique therapeutic challenges due to their heterogeneity and poor prognosis to standard treatments. Particle therapy offers improved dose distribution and potentially higher relative biological effectiveness, however, its immunological effects in patients remain poorly understood. Investigating peripheral immune cell changes could offer valuable insights for integrating immunotherapies and optimizing treatment outcomes.

**Methods:**

In this observational study, we enrolled 12 patients with head and neck bone and soft tissue tumors treated at our center between November 1, 2022, and November 1, 2024. Treatment modalities included proton or carbon-ion radiotherapy, with or without chemotherapy, targeted therapy, or immunotherapy. Peripheral blood samples were collected both before and after the completion of radiotherapy. Hematologic assessments were conducted, including total lymphocyte counts and immunophenotyping of CD3+, CD4+, CD8+, and other lymphocyte subsets. Statistical analyses, including paired Student’s t-test, Wilcoxon signed-rank tests and univariate analysis, were performed to investigate associations between lymphocyte changes and clinical factors.

**Results:**

Minor reductions were noted in CD3^+^ and CD4^+^ T cell subsets, accompanied by a small increase in CD3^+^CD4^–^CD8^–^ subsets. Even after excluding the patient who received immunotherapy, the observed trend in lymphocyte counts and subset changes remained consistent. This finding suggests that, compared with conventional photon radiotherapy, particle therapy may better preserve immune function. Remarkably, all patients were alive and showed no evidence of disease progression during the study period.

**Conclusion:**

Particle therapy in patients with head and neck bone and soft tissue tumors induces modest immunological alterations, suggesting it may preserve immune function more effectively than conventional photon radiotherapy. These preliminary findings from our small cohort support further research into combining particle therapy with immunomodulatory strategies, potentially enhancing clinical outcomes and expanding therapeutic options for these challenging malignancies.

## Introduction

Bone and soft tissue tumors are rare malignancies marked by significant histological and pathological heterogeneity (1). This diversity leads to considerable variations in disease progression, treatment responses, and clinical outcomes, making their management highly challenging. Despite advances in oncology, the overall prognosis for these tumors remains poor, underscoring the urgent need for more effective treatment strategies (2). Radiotherapy plays a pivotal role in the multidisciplinary management of bone and soft tissue tumors. Systemic therapies, such as surgery, chemotherapy and targeted agents, are also needed depending on characteristics of each cases, while sometimes limited due to the genetic and biological variability (3).

However, conventional radiotherapy faces significant limitations. Certain tumor subtypes exhibit inherent radioresistance, and radiation close to sensitive anatomical regions could result in severe toxicities. These challenges highlight the need for advanced radiotherapy techniques. Particle therapy, including proton and carbon-ion radiotherapy, has emerged as a promising approach for treating bone and soft tissue tumors (4, 5). Its unique physical properties, such as the Bragg peak, allow for precise dose delivery that maximizes tumor irradiation while sparing surrounding healthy tissue. Moreover, carbon-ion therapy offers a higher relative biological effectiveness (RBE), which can overcome radioresistance in certain tumors (6). Clinical experiences over the past few decades have demonstrated that carbon-ion radiotherapy can improve local control rates and reduce treatment-related toxicity, making it an attractive option for these complex malignancies (7).

Integrating particle therapy into clinical practice holds significant potential for enhancing patient outcomes and quality of life. Radiotherapy exerts its antitumor effects primarily through direct DNA damage and the induction of oxidative stress. In recent years, increasing interest has arisen in the connection between radiotherapy and the immune system (8). Lymphocytes, especially T cells, are central to immune responses. CD4+ helper T cells and CD8+ cytotoxic T cells have distinct but complementary roles in cellular immunity. Peripheral blood analyses provide a snapshot of lymphocyte dynamics, while more detailed examinations can reveal changes in specific lymphocyte subsets and their functional states (9). Understanding these changes is crucial for assessing immune status during and after radiotherapy, which can inform treatment planning and optimization.

Radiotherapy can modulate the tumor microenvironment in complex and paradoxical ways. On one hand, it can induce immunogenic cell death, release tumor antigens, and activate dendritic cells, thereby stimulating antitumor immune responses. On the other hand, radiotherapy can cause lymphopenia and suppress certain immune functions, depending on the dose and the irradiated volume (10). Several studies have shown that radiation-induced lymphopenia is strongly correlated with poor survival and increased metastasis rates. B cells are identified as the most radiosensitive lymphocyte subpopulation, followed by T cells and NK cells, with significant implications for treatment planning and immune response (11). Particle therapies like proton and carbon-ion therapy, due to their precise dose distribution, may reduce lymphocyte damage compared to traditional photon therapy.

Particle therapy has garnered attention for its potential immunomodulatory effects. Emerging studies suggest that carbon-ion radiation may induce stronger immunogenic cell death and elicit more robust antitumor immune responses compared to conventional photon therapy (12). For instance, research involving prostate cancer patients has reported alterations in immune parameters following carbon-ion irradiation, showing that overall frequencies of major immune cell types (CD3+, CD4+, CD8+ T cells, NK cells) remained unchanged, and there was an increase in the CD4/CD8 ratio, lymphocyte proliferation, and T-cell functionality, along with a reduction in B cells, offering insights into the complex interactions between particle therapy and the immune system (13). However, there is a paucity of research focusing specifically on the impact of particle therapy on peripheral immune cell dynamics in patients with head and neck bone and soft tissue tumors.

In this study, we analyzed peripheral blood lymphocyte data from patients with head and neck bone and soft tissue tumors treated at our center. By evaluating the effects of particle therapy on the immune system across different patient profiles, we aim to identify potential variations in treatment responses and to explore strategies for optimizing therapeutic approaches. Our findings, though preliminary and from a small cohort, may contribute to the development of novel treatment paradigms that combine particle therapy with immune-based interventions, enhance understanding of the immunological effects induced by particle radiation, and help improve outcomes of these challenging malignancies.

## Method

### Patient selection

An observational study was conducted on patients with bone and soft tissue tumors at the Shanghai Proton and Heavy Ion Center (SPHIC) between November 1, 2022, and November 1, 2024. A total of 12 patients were included in the study, as detailed in **Table 1**. The study received approval from the Institutional Review Board (Ethics Committee approval number: SPHIC-TR-HNCNS-2022-63), and written informed consent was obtained from all participants. This was designed as a purely observational study without any interventional component; the treatment decisions were based solely on clinical considerations and were not influenced by this research protocol. All patients underwent thorough medical evaluations and were staged based on the 8th edition of the American Joint Committee on Cancer (AJCC/UICC) staging system. Treatment approaches included proton radiotherapy (PRT), carbon-ion radiotherapy (CIRT), chemotherapy, targeted therapy, and immunotherapy, according to the specific clinical characteristics of each patient. Particle radiotherapy was delivered according to institutional protocols. Carbon ion therapy was administered at doses ranging from 63–72 GyE with fraction sizes of 3.0-4.0 GyE, while proton therapy was delivered at 60-70.4 GyE with fraction sizes of 2.0-2.2 GyE. Treatment planning incorporated RBE considerations, with a variable RBE value of 2.0-3.0 depending on each patient used for carbon ions and 1.1 for protons. The selection of beam modality, total dose, and fractionation was determined based on tumor histology, anatomical location, proximity to critical structures, and patient-specific factors.

**Table 1:** Patient, tumor and treatment characteristics.

Patient No.	Gender	Age	Pathology	Disease status	Radiation status	Target volume	Localization	Beamline	Dose *Gy (RBE)*	Fx	Fraction Dose	Surgery	Other Treatments
1	Male	49	LPS	Primary	Initial	159.64	Neck	C	63	18	3.5	Y	None
2	Female	44	CS	Primary	Initial	61.92	Skull Base	C	70	20	3.5	Y	None
3	Male	34	CS	Primary	Initial	115.84	Skull Base	P	70.4	32	2.2	Y	None
4	Male	37	CS	Primary	Initial	81.5	Skull Base	P	70.4	32	2.2	Y	None
5	Male	56	CS	Recurrent	Initial	95.23	Skull Base	P	70.4	32	2.2	Y	None
6	Male	18	MPNST	Primary	Initial	255.74	Skull Base	C	72	18	4	Y	Chemo
7	Male	61	Ch	Primary	Initial	43.55	Skull Base	P	70.4	32	2.2	Y	None
8	Male	35	Ch	Primary	Initial	56.02	Skull Base	P	70.4	32	2.2	Y	none
9	Male	70	Ch	Recurrent	Initial	124.23	Skull Base	P	70.4	32	2.2	Y	None
10	Male	37	Ch	Primary	Initial	91.33	Skull Base	P	70.4	32	2.2	Y	None
11	Female	27	RMS	Primary	Initial	53.06	Skull Base	P	60	30	2	Y	Target
12	Male	56	INI	Recurrent	Re-RT	140.64	Nasal Sinus	C	63	21	3	Y	IM

LPS, Liposarcoma; CS, Chondrosarcoma; MPNST, Malignant Peripheral Nerve Sheath Tumor; Ch, Chordoma; RMS, Rhabdomyosarcoma; INI, INI1-deficient tumors; C, carbon-ion radiation; P, proton radiation; Y, had surgery; Chemo, chemotherapy; Target, target therapy; IM, immunotherapy.

### Blood collection and peripheral hematologic assessments

Peripheral blood samples (collected in EDTA tubes) were obtained from all patients before the first fraction of radiation and after the final fraction of radiation. All samples were processed within 2 hours of collection according to standardized protocols. Hematologic assessments, including detailed immunophenotyping, were performed by the hospital’s clinical laboratory department using a BD FACSCanto II flow cytometer (BD Biosciences, San Jose, CA) following standardized institutional protocols. For immunophenotyping, a comprehensive antibody panel was used to evaluate multiple lymphocyte subsets, including T cells (CD3+), helper T cells (CD3+CD4+), cytotoxic T cells (CD3+CD8+), double-negative T cells (CD3+CD4-CD8-), and PD-1 expressing T cell subsets. All analyses were conducted by professional laboratory staff following the hospital’s standardized management regulations, ensuring objectivity, independence, and reproducibility of results.

All patients underwent routine follow-up evaluations at 1, 3, and 6 months after the completion of radiotherapy, including magnetic resonance (MR) scans. Assessments included monitoring for recurrence and evaluating survival outcomes. Due to practical challenges in standardizing blood sample collection for flow cytometry testing during follow-up periods, longitudinal immune profiling was not included in the current analysis.

### Statistical analysis

Statistical analyses were performed to assess changes in lymphocyte counts before and after radiation therapy. Normality of data distribution was assessed using Shapiro-Wilk tests. Both parametric (paired Student’s t-test) and non-parametric (Wilcoxon signed-rank test) methods were applied to determine statistical significance, with a p-value threshold set at ≤ 0.05. For comparing baseline differences in lymphocyte subsets between pathology types, Kruskal-Wallis tests were used, while Mann-Whitney U tests were employed to compare primary versus recurrent disease status groups.

Two types of change metrics were calculated for immunological parameters: (1) “Mean absolute change” representing the difference in percentage points (post-radiotherapy value minus pre-radiotherapy value) and (2) “Percentage change” representing the relative change calculated as [(post-value - pre-value)/pre-value] × 100%.

Univariate analyses were conducted to explore associations between lymphocyte changes and relevant clinical factors, including pathology type (chondrosarcoma, chordoma, others), disease status (primary vs. recurrent), beam modality (carbon-ion vs. proton), dosage and other treatment parameters. For these analyses, percentage changes in lymphocyte subsets were compared between groups. Data analysis and graphical representations were generated using GraphPad Prism (version 9.0; GraphPad Software, La Jolla, CA). Additional statistical analyses were carried out using R (version 4.4.1; RStudio 2024.09.1 + 394).

## Results

### Clinical characteristics

A total of 12 patients with bone or soft-tissue tumors were included in the study, with detailed information provided in **Table 1**. The median age at diagnosis was 40.5 years (range: 18–70 years). All primary tumor sites were in the head and neck region, including one patient with a neck tumor, one with a nasal sinus tumor, and 10 with tumors in the skull base. The cohort consisted of four patients with chondrosarcoma, four with chordoma, and four with liposarcoma, peripheral neurinoma, rhabdomyosarcoma, and INI-deficient tumors. Nine patients had primary tumors, while three had recurrent tumors. Eight patients received proton beam therapy, and four were treated with carbon-ion radiotherapy (CIRT). The selection of beam modality and treatment dose was determined based on tumor pathology and anatomical location. Nine patients underwent particle therapy alone, while three received additional therapies—chemotherapy, targeted therapy, and immunotherapy, respectively—all administered prior to the initiation of radiation therapy.

### Follow-up and oncological outcomes

The median follow-up duration was 10.5 months (range: 2–16 months). At the latest follow-up, all 12 patients remained in contact, were alive, and showed no signs of recurrence. However, one patient with a recurrent INI-deficient tumor developed necrosis in the target area following CIRT. While these short-term outcomes are encouraging, the limited follow-up period prevents definitive conclusions about long-term tumor control and survival. Detailed outcome information is presented in **Table 2**, including Overall Survival (OS), Local Control (LC), and Distant Progression-Free Survival (DPFS) data.

**Table 2:** Clinical outcomes of bone and soft tissue tumors.

Patient no.	Patient status	Follow-up interval	OS	LC	DPFS
1	alive	11	11	11	11
2	alive	16	16	16	16
3	alive	15	15	15	15
4	alive	10	10	10	10
5	alive	8	8	8	8
6	alive	3	3	3	3
7	alive	13	13	13	13
8	alive	13	13	13	13
9	alive	12	12	12	12
10	alive	9	9	9	9
11	alive	2	2	2	2
12	alive	7	7	7	7

OS, Overall Survival (months); LC, Local Control (months); DPFS, Distant Progression-Free Survival (months).

### Hematologic effects

**Figure 1:**
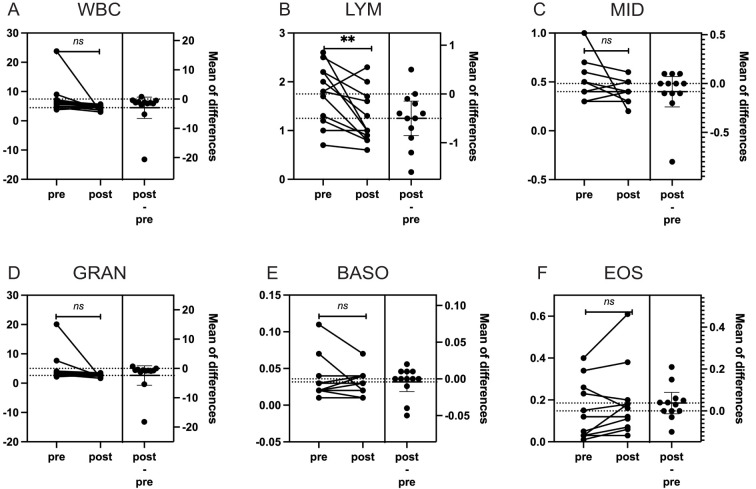
The impact of particle radiotherapy on peripheral blood cells in patients with gead and neck bone and soft Tissue tumors. (*p<0.05, ns, not significant). Pre, pre-radiotherapy; Post, post-radiotherapy); **(A)** Changes in the count of White Blood Cells (WBC), **(B)** Changes in the count of Lymphocytes (LYM), **(C)** Changes in the count of Middle Cells (MID), which include monocytes, immature granulocytes, etc, **(D)** Changes in the count of Granulocytes (GRAN), **(E)** Changes in the count of Basophils (BASO), **(F)** Changes in the count of Eosinophils (EOS).

To assess the effects of radiation on peripheral blood, complete blood count (CBC) data from the 12 patients were analyzed. CBCs collected before the initiation of radiotherapy and after its completion were compared. After confirming non-normal distribution using Shapiro-Wilk tests, changes in circulating immune cells, including white blood cells (WBCs), neutrophils, lymphocytes, monocytes, eosinophils, and basophils, were evaluated using paired Student’s t-test (**Figure 1**). Lymphocyte counts showed a significant decrease (p = 0.01), indicating a specific impact of radiation on the immune system in patients with bone or soft-tissue sarcoma. This decrease, however, was modest compared to the substantial lymphocyte reductions reported in conventional photon radiotherapy (14). To address potential confounding effects of immunotherapy, the analysis was repeated after excluding the immunotherapy patient, and the results remained consistent (p = 0.008).

### Effects on lymphocyte subtypes

To investigate the immunomodulatory effects of particle therapy on head and neck bone and soft-tissue tumors, we analyzed peripheral blood lymphocyte subsets before and after therapy. First, we assessed whether there were any baseline differences in pre-treatment lymphocyte subsets between different pathology types or disease status groups. This analysis revealed no statistically significant differences in any lymphocyte subpopulation between pathology groups (chondrosarcoma, chordoma, and others) or between primary and recurrent tumors (all p-values > 0.05), suggesting comparable immune profiles at baseline across our patient cohort.

**Figure 2:**
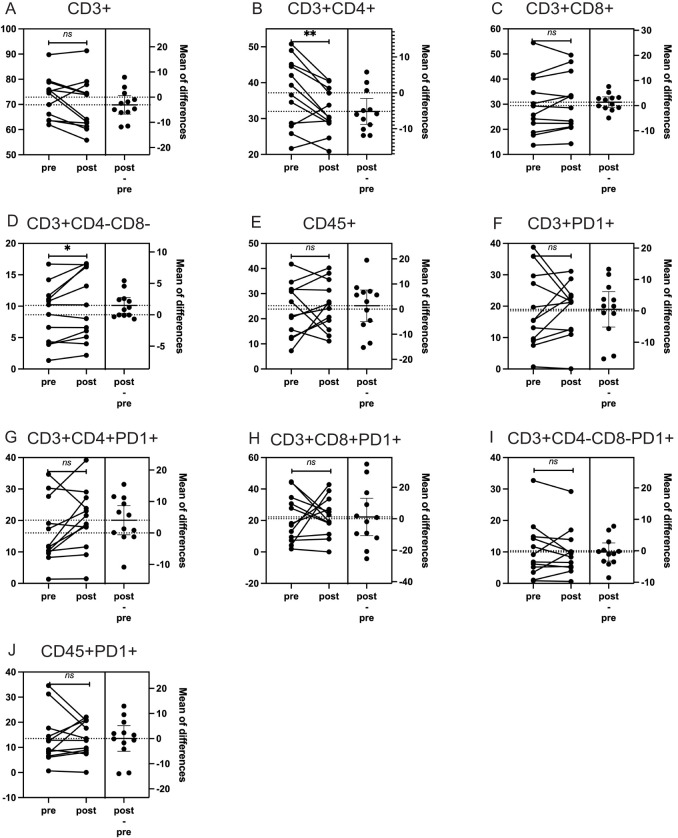
The impact of particle radiotherapy on immune cell subsets in patients with head and neck bone and soft tissue tumors. (*p<0.05, **p<0.01, ns, not significant. Pre, pre-radiotherapy; Post, post-radiotherapy). **(A)** Changes in the proportion of CD3+ T cells, **(B)** Changes in the proportion of CD3+CD4+ helper T cells. **(C)** Changes in the proportion of CD3+CD8+ cytotoxic T cells, **(D)** Changes in the proportion of CD3+CD4-CD8- double-negative T cells, **(E)** Changes in the proportion of CD45+ leukocytes, **(F)** Changes in the proportion of CD3+PD1+ T cells expressing PD-1, **(G)** Changes in the proportion of CD3+CD4+PD1+ helper T cells expressing PD-1, **(H)** Changes in the proportion of CD3+CD4-CD8-PD1+ double-negative T cells expressing PD-1, **(I)** Changes in the proportion of CD3+CD8+PD1+ cytotoxic T cells expressing PD-1, **(J)** Changes in the proportion of CD45+PD1+ leukocytes expressing PD-1.

We then analyzed changes in the percentages of CD3+, CD3+CD4+, CD3+CD8+, CD3+CD4-CD8-, CD45+, CD3+PD1+, CD3+CD4+PD1+, CD3+CD8+PD1+, CD3+CD4-CD8-PD1+, and CD45+PD1+ subgroups before and after treatment. Using both paired t-tests and Wilcoxon signed-rank tests, we identified significant findings that were consistent across both statistical methods: an 11.6% decrease in CD3+CD4+ lymphocytes (t-test: p = 0.009; Wilcoxon: p = 0.012) and a 21.4% increase in CD3+CD4-CD8- lymphocytes (t-test: p = 0.024; Wilcoxon: p = 0.064). Other subgroups did not show significant changes with either statistical method (**Figure 2**, **Table 3**).

**Table 3:** Summary of changes in lymphocyte subpopulations in H&N bone and soft-tissue patients before and after RT.

	CD3+	CD3+CD4+	CD3+CD8+	CD3+ CD4-CD8-	CD45+	CD3+PD1+	CD3+CD4+ PD1+	CD3+CD8+ PD1+	CD3+CD4-CD8-PD1+	CD45+PD1+
Mean	-3.054	-5.186	1.37	1.497	1.344	0.4917	4.061	1.167	-0.3808	0.0475
SD	5.787	5.672	3.375	1.978	9.882	8.884	7.282	18.67	4.549	8.053
SEM	1.67	1.637	0.9744	0.5709	2.853	2.565	2.102	5.389	1.313	2.325
95%CI	-6.731 to 0.6224	-8.789 to -1.582	-0.7746 to 3.515	0.2402 to 2.753	-4.934 to 7.623	-5.153 to 6.136	-0.5658 to 8.687	-10.70 to 13.03	-3.271 to 2.509	-5.069 to 5.164
R2	0.2331	0.477	0.1523	0.3846	0.01979	0.00333	0.2533	0.004242	0.007589	0.00003796
p-t	0.0947	**0.009**	0.1873	**0.0238**	0.6467	0.9841	0.0795	0.8326	0.7772	0.9841
p-t*	ns	**	ns	*	ns	ns	ns	ns	ns	ns
p-w	0.0923	**0.0122**	0.2334	0.0640	0.5693	0.6772	0.0771	0.9697	0.4697	0.9097
p-w*	ns	*	ns	ns	ns	ns	ns	ns	ns	ns
Percentage Change	-4.271818	-11.551531	6.06747698	21.3706731	27.3511558	13.106263	38.1011446	77.6850142	31.8939441	12.9899452
	CD3+	CD3+CD4+	CD3+CD8+	CD3+ CD4-CD8-	CD45+	CD3+PD1+	CD3+CD4+ PD1+	CD3+CD8+ PD1+	CD3+CD4-CD8-PD1+	CD45+PD1+
Mean	-4.052	-5.922	1.018	1.694	0.8582	0.5927	4.418	1.443	-0.3945	0.1064
SD	4.868	5.314	3.301	1.947	10.21	9.311	7.526	19.56	4.77	8.443
SEM	1.468	1.602	0.9954	0.587	3.079	2.807	2.269	5.896	1.438	2.546
95%CI	-7.322 to -0.7816	-9.492 to -2.352	-1.200 to 3.236	0.3858 to 3.001	-6.003 to 7.719	-5.662 to 6.848	-0.6378 to 9.474	-11.69 to 14.58	-3.599 to 2.810	-5.566 to 5.778
R2	0.4325	0.5774	0.09472	0.4543	0.007708	0.004438	0.2749	0.005952	0.007468	0.0001745
p-t	**0.0201**	**0.0041**	0.3305	0.0162	0.7861	0.837	0.0801	0.8116	0.7894	0.9675
p-t*	*	**	ns	*	ns	ns	ns	ns	ns	ns
p-w	**0.0186**	**0.0098**	0.4131	0.0420	0.7646	0.7002	0.0830	0.9658	0.5771	0.8984
p-w*	*	**	ns	*	ns	ns	ns	ns	ns	ns
Percentage Change	-5.689472	-13.82133	5.476706951	24.01437173	25.02986306	22.71022448	40.69590104	93.64781175	37.25328657	22.43531217

Mean, Mean of difference; represents absolute change in percentage points; SD, standard deviation; SEM, standard error of mean; 95%CI, 95% confidence interval; R2, coefficient of determination; Percentage Change represents relative change [(post-pre)/pre]×100%. p, p-value; p-t, p-value based on paired t-test; p-w, p-value based on Wilcoxon signed-rank test; p*: significance level ( *p<0.05, **p<0.01, ns, not significant); Bold values represent statistically significant differences (p ≤ 0.05). Including (table above) and excluding (table below) the patient who received immunotherapy.

When compared to published data from conventional photon radiotherapy, where CD3+ and CD4+ T cell populations typically decrease more than 50% (15), our observed changes were substantially smaller (5.7% decrease in CD3+ and 11.6% decrease in CD3+CD4+), supporting the hypothesis that particle therapy may have a more moderate impact on lymphocyte populations (**Table 3**).

**Figure 3:**
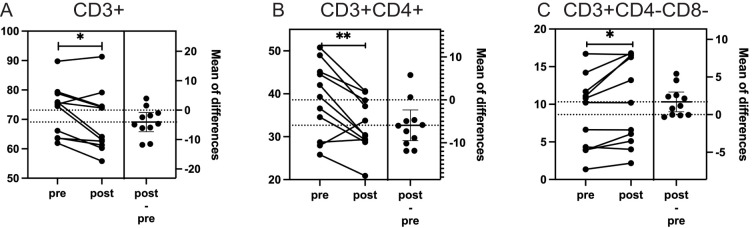
The impact of particle radiotherapy on significantly changed immune cell subsets in patients with head and neck bone and soft tissue tumors excluding the patient who received immunotherapy. (*p<0.05, **p<0.01; Pre, pre-radiotherapy; Post, post-radiotherapy). **(A)** Changes in the proportion of CD3+ T cells (p = 0.02), **(B)** Changes in the proportion of CD3+CD4+ helper T cells (p = 0.011), **(C)** Changes in the proportion of CD3+CD4-CD8- double-negative T cells (p = 0.029).

Excluding the immunotherapy patient revealed additional findings, including a 5.7% decrease in CD3+ lymphocytes (p = 0.02), along with the previously observed changes in CD3+CD4+ and CD3+CD4-CD8- lymphocytes. Detailed results for all patients and for the cohort excluding the immunotherapy patient are presented in **Figure 3**; **Table 3**. We also explored the results after excluding targeted therapy and chemotherapy patients, which were basically consistent with those shown in **Table 3** (data not shown).

### Univariate analysis of lymphocyte subgroup changes

Exploratory univariate analyses were conducted to evaluate potential associations between clinical factors (including pathology type, treatment status, beam modality, tumor location, total dose, fraction dose, age, gender, and target volume) and changes in lymphocyte subpopulations. The analysis revealed that changes in CD3+CD4-CD8- lymphocytes were influenced by pathology type (p = 0.026), with chondrosarcoma patients showing more pronounced increases (46.5% ± 16.7%) compared to chordoma (10.0% ± 16.9%) and other tumor types (7.63% ± 21.2%). Additionally, alterations in CD3+CD8+ lymphocytes were associated with tumor recurrence status (p = 0.049), with recurrent tumors showing greater increases (19.8% ± 8.89%) than primary tumors (1.48% ± 7.40%). We also performed analyses to examine potential associations between total dose, fraction size and changes in lymphocyte subpopulations. No statistically significant correlations were identified between these dosimetric factors and the magnitude of change in any lymphocyte subset (all p-values > 0.05).

It is important to note that these findings should be interpreted with caution due to the small sample size. Many variables could not undergo meaningful statistical evaluation due to substantial disparities in sample sizes after grouping and were therefore excluded from the analysis. Detailed results of these exploratory analyses are presented in **Table 4**, with significant associations highlighted.

**Table 4:** Summary of unitivariable analyses of clinical factors.

Subsets	Beamline			Pathology				Disease status		
	C	P	P. overall	CS	Ch	Others	P. overall	Initial	Re	P. overall
	N=4	N=8		N=4	N=4	N=4		N=9	N=3	
CD3	-5.52 (12.8)	-3.65 (5.23)	0.795	-6.68 (6.18)	-4.94 (6.98)	-1.20 (11.1)	0.652	-7.37 (5.74)	5.04 (6.40)	0.055
CD3CD4	-9.22 (20.2)	-12.72 (14.1)	0.77	-18.43 (5.83)	-18.26 (3.92)	2.03 (21.6)	0.088	-13.23 (15.3)	-6.51 (18.3)	0.607
CD3CD8	0.81 (8.10)	8.70 (11.9)	0.211	9.93 (7.40)	9.63 (14.0)	-1.35 (9.53)	0.284	1.48 (7.40)	19.8 (8.89)	**0.04**9*
CD3CD4-CD8-	21.5 (30.4)	21.3 (24.1)	0.994	46.5 (16.7)	10.0 (16.9)	7.63 (21.2)	0.026*	20.7 (23.8)	23.3 (33.9)	0.91
CD45	9.18 (34.1)	36.4 (103)	0.513	-7.15 (33.2)	16.3 (49.7)	72.9 (135)	0.428	20.2 (97.5)	48.8 (24.2)	0.437
CD3PD1	29.5 (89.0)	4.89 (43.3)	0.63	19.8 (33.6)	12.0 (53.5)	7.58 (93.5)	0.965	20.5 (50.4)	-9.01 (90.0)	0.634
CD3CD4PD1	48.9 (37.0)	32.7 (57.6)	0.572	19.6 (21.7)	61.8 (67.4)	32.9 (55.5)	0.526	35.9 (45.5)	44.7 (75.0)	0.862
CD3CD8PD1	243 (414)	-4.73 (54.7)	0.318	230 (403)	-20.15 (47.7)	23.6 (143)	0.358	122 (279)	-54.58 (38.1)	0.099
CD3CD4-CD8-PD1	34.2 (103)	30.8 (107)	0.959	-12.30 (12.1)	79.3 (141)	28.7 (106)	0.478	16.2 (74.7)	79.1 (170)	0.591
CD45PD1	32.0 (106)	3.48 (43.5)	0.636	55.6 (80.2)	3.23 (51.4)	-19.88 (57.2)	0.284	21.6 (63.5)	-12.88 (83.8)	0.563

C, carbon-ion radiation; P, proton radiation; CS, Chondrosarcoma; Ch, Chordoma; Others, other tumors; Initial, patients with initial radiotherapy; Re, recurrent tumors. Values are presented as mean percentage change ± standard deviation (SD). Bold * values indicate statistically significant differences (p < 0.05).

## Discussion

Radiotherapy exerts diverse effects on immune cells across different tumor types. Previous studies figured out that in head and neck cancers, photon radiotherapy significantly reduces peripheral CD4^+^ and CD8^+^ T lymphocyte counts post-treatment, with reductions typically ranging more than 50% (15). In contrast, our findings suggest that particle therapy induces substantially smaller changes in lymphocyte populations (5.7% decrease in CD3+ and 11.6% decrease in CD3+CD4+). This comparison, while requiring validation in direct comparative studies, suggests that particle therapy may have a more moderate impact on lymphocyte populations than conventional photon radiotherapy. Several factors might contribute to this difference, including the more precise dose distribution of particle therapy and its unique physical properties.

In breast cancer patients, hypofractionated radiotherapy causes less pronounced reductions in peripheral lymphocyte counts compared to conventional fractionation, indicating that fractionation schemes have different influence immune modulation. In esophageal and lung cancers, radiotherapy can stimulate lymphocyte proliferation and activation, thereby strengthening antitumor immunity (14, 16). Conversely, in low-immunogenic tumors such as pancreatic cancer, radiotherapy may not significantly improve immune cell counts or function (17).

Analyzing lymphocyte subsets based on surface markers and functional roles provides critical insights into immune changes. CD4^+^ T cells (helper T cells) regulate immune responses and activate other immune cells, while CD8^+^ T cells (cytotoxic T cells) directly kill tumor cells and are pivotal for antitumor immunity (18). Radiotherapy induces distinct immune cell changes in peripheral blood and tumor tissue. Peripheral lymphocytes, particularly CD4^+^ T cells and B cells, are highly radiosensitive, often showing significant reductions post-treatment. In contrast, the number of tumor-infiltrating lymphocytes (TILs), especially CD8^+^ T cells, frequently increases within tumors, indicating a localized activation of immune responses (11). This apparent disparity may result from radiotherapy-induced damage to circulating immune cells coupled with the recruitment of immune cells to tumor sites. These dynamics highlight the complex and variable impact of radiotherapy on systemic and local immunity. In our cohort, we observed modest decreases in circulating CD3+ and CD4+ T cells following particle therapy. However, without direct assessment of the tumor microenvironment, we cannot determine whether particle therapy induces similar localized immune activation inside tumor as observed with photon therapy (16).

Several studies have reported that hypofractionated radiotherapy and SBRT could reduce the number of treatment sessions and the irradiated volume, mitigating damage to normal tissues and circulating immune cells (19). The relationship between radiotherapy dose-fractionation parameters and immune modulation remains an important area for investigation (11, 20). In our study, radiation dose was relatively consistent, ranging from 63 Gy (RBE) to 72 Gy (RBE), with dose per fraction from 2.0 Gy (RBE) to 3.5 Gy (RBE). Thus, as shown in our results, neither the total dose, fractional dose, nor the number of fractions had impact on the immune subgrouping, suggesting that within this dose range, the immunomodulatory effects were relatively consistent. However, given our small sample size, these analyses have limited power and future studies with larger cohorts and more diverse fractionation schemes will be needed to elucidate optimal particle therapy parameters.

Different radiation modalities, including photons, protons, and carbon ions, cause varying degrees of immune cell damage (18, 19). Combination therapies, such as chemotherapy and surgery, can exacerbate immune suppression, further altering lymphocyte subset dynamics. Additionally, patient-specific factors, including age, sex, tumor type and stage, baseline immune function, and comorbidities, all contribute to the effects of radiotherapy on lymphocytes, which makes the modulation of immune cells very complex (20). In this study, the small sample size and short follow-up period limit our statistical approach and the generalizability of our findings. Additional cases and extended follow-up durations are needed to further validate the results and strengthen the conclusions.

Most studies evaluate immune cell changes shortly after radiotherapy, typically within one month, limiting insights into long-term effects. Our study shares this limitation, due to the challenges of standardizing blood sample collection for flow cytometry testing during follow-up periods for each patient. Continuous monitoring over extended follow-up intervals, such as three, six, and twelve months, would provide a more comprehensive understanding of immune recovery dynamics. Some studies have indicated that CD4^+^ T cells, being highly radiosensitive, may take months to return to baseline levels, whereas CD8^+^ T cells recover more quickly (11). Unveiling these recovery patterns is crucial for optimizing the timing of immunotherapy to enhance efficacy and minimize adverse effects.

Radiotherapy can also modulate PD-1/PD-L1 expression on tumor and immune cells. By inducing DNA damage and activating downstream signaling pathways, radiotherapy may upregulate PD-L1 expression, allowing tumor immune evasion (14). This underlines the potential of combining radiotherapy with immune checkpoint inhibitors, such as anti-PD-1/PD-L1 antibodies, to enhance antitumor immunity. Understanding these interactions informs the design of optimized combination therapy regimens. In our small cohort, we observed that PD-1 expression on peripheral blood lymphocytes did not significantly increase after particle radiotherapy. This suggests that particle radiation may have minimal impact on PD-1-dependent immune suppression in the peripheral circulation, though we cannot draw conclusions about changes within the tumor microenvironment. This observation, albeit preliminary, potentially aligns with the generally low response rates of soft tissue sarcomas to immune checkpoint blockade therapies. Our findings suggest that combining immune-activating strategies may be necessary to enhance therapeutic outcomes in these tumor types (21).

Interestingly, we noted a significant increase in the CD3^+^CD4^-^CD8^-^ subset, which highlights the complex immunological landscape of sarcomas. This population includes not only cytotoxic immune cells but also inflammation-associated immune cells, indicating an intricate interplay within the tumor microenvironment (22). The biological significance of this increase in double-negative T cells following particle therapy requires further investigation. To better understand the underlying mechanisms of this phenomenon and confirm the patterns observed in our limited sample, more detailed analyses of lymphocyte subsets in larger patient cohorts are warranted.

Insights into radiotherapy-induced immune modulation, such as those suggested by our preliminary findings, may eventually guide personalized treatment strategies. Although our study is too limited to provide definitive guidance, future research with larger cohorts could help determine whether adjusting particle therapy parameters based on individual immune profiles could help preserve immune function. Monitoring immune cell dynamics during and after treatment, as we have initiated in our expanded protocol, may allow for timely interventions to mitigate immune suppression and promote recovery.

Conclusion: This study provides preliminary evidence from a small cohort of patients that particle therapy induces relatively minor alterations in peripheral immune cell profiles in patients with head and neck bone and soft tissue tumors. The overall immune landscape remained relatively stable compared to what has been reported with photon therapy. From a clinical perspective, understanding the immunomodulatory effects of particle therapy could potentially guide the development of more personalized treatment strategies. While our current data are too limited to change clinical practice, they provide rationale for further investigation. Large-scale, multicenter studies with extended follow-up periods are essential to validate these preliminary results and determine their clinical significance.

Despite the limitations of our study, our findings contribute to the growing evidence that particle therapy may offer immunological advantages for patients with head and neck bone and soft tissue tumors. Additionally, the potential to combine particle therapy with immunotherapy warrants further exploration and could contribute to the development of more effective treatment paradigms for these challenging malignancies.

## Data Availability

The original contributions presented in the study are included in the article/supplementary material. Further inquiries can be directed to the corresponding author.
