# Correction: Changes in lymphocyte subsets pre- and post-particle radiotherapy in head and neck bone and soft tissue tumors

**DOI:** 10.3389/fonc.2025.1648468

**Published:** 2025-06-27

**Authors:** Haojiong Zhang, Jing Gao, Jiyi Hu, Weixu Hu, Qingting Huang, Lin Kong

**Affiliations:** ^1^ Department of Radiation Oncology, Shanghai Proton and Heavy Ion Center, Fudan University Cancer Hospital, Shanghai, China; ^2^ Shanghai Key Laboratory of Radiation Oncology, Shanghai, China; ^3^ Shanghai Engineering Research Center of Proton and Heavy Ion Radiation Therapy, Shanghai, China

**Keywords:** bone and soft tissue tumors, head and neck cancer, peripheral lymphocyte immunophenotype, particle radiation, radiation immunity

Affiliation “Department of Radiation Oncology, Shanghai Proton and Heavy Ion Center, Fudan University Cancer Hospital, Shanghai, China” was omitted for all authors. This affiliation has now been added for all authors.

Affiliation “Shanghai Key Laboratory of Radiation Oncology, Shanghai, China” was omitted for all authors. This affiliation has now been added for all authors.

Affiliation “Shanghai Engineering Research Center of Proton and Heavy Ion Radiation Therapy, Shanghai, China” was omitted for all authors. This affiliation has now been added for all authors.

All authors were erroneously assigned to affiliation Shanghai Proton and Heavy Ion Center (SPHIC), Shanghai, China. This affiliation has now been removed for all authors.

The original version of this article has been updated.

